# Transgenesis in parasitic helminths: a brief history and prospects for the future

**DOI:** 10.1186/s13071-022-05211-z

**Published:** 2022-03-28

**Authors:** M. J. Quinzo, M. J. Perteguer, P. J. Brindley, A. Loukas, J. Sotillo

**Affiliations:** 1 Parasitology Reference and Research Laboratory, Centro Nacional de Microbiología, Instituto de Salud Carlos III, Majadahonda, Madrid, Spain; 2 Escuela Internacional de Doctorado, Universidad Nacional de Educación a Distancia (UNED), Madrid, Spain; 3 Department of Microbiology, Immunology and Tropical Medicine, and Research Center for Neglected Diseases of Poverty, School of Medicine and Health Sciences, George Washington University, Washington, DC 20037 USA; 4 Centre for Molecular Therapeutics, Australian Institute of Tropical Health and Medicine, James Cook University, Cairns, QLD Australia

**Keywords:** Helminths, Genetic edition, Transgenesis, CRISPR, RNAi

## Abstract

Helminth infections impact the health of hundreds of millions of persons globally and also cause important economic losses in livestock farming. Methodological limitations as well as the low attention given to the study of helminths have impacted biological research and, thus, the procurement of accurate diagnosis and effective treatments. Understanding the biology of helminths using genomic and proteomic approaches could contribute to advances in understanding host–helminth interactions and lead to new vaccines, drugs and diagnostics. Despite the significant advances in genomics in the last decade, the lack of methodological adaptation of current transgenesis techniques has hampered the progression of post-genomic research in helminthology. However, the application of new techniques, such as CRISPR, to the study of trematodes and nematodes has opened new avenues for genome editing-powered functional genomics for these pathogens. This review summarises the historical advances in functional genomics in parasitic helminths and highlights pending limitations that will need to be overcome to deploy transgenesis tools.

**Graphical Abstract:**

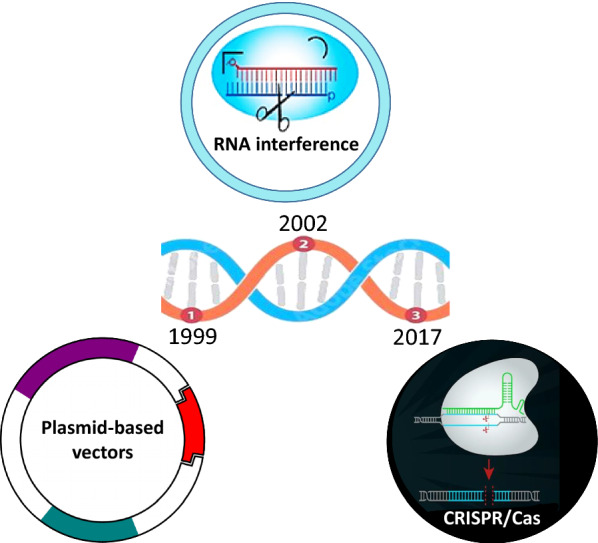

**Supplementary Information:**

The online version contains supplementary material available at 10.1186/s13071-022-05211-z.

## Background

Approximately one sixth of the human population is infected with parasitic helminths [1, 2], mainly in countries in tropical and subtropical regions, causing growth delay and cognitive development disruption in children as well as significant morbidities and chronic disabilities in adults [3, 4]. Globally, helminth infections have been estimated to have resulted in approximately 14 million disability-adjusted life years (DALYs) lost yearly during the last decade [1]. In addition, the estimated annual cost in Europe associated with helminth infections in livestock (gastrointestinal nematodes, common liver flukes and bovine lungworm) is close to 2 billion euros [5]. Despite the medical and economical importance of helminthiases, the study of these diseases receives less than 1% of the global research budget, and most are still considered as neglected tropical diseases [5].

The most common human diseases are caused by soil-transmitted helminths (STHs) causing ascariasis, trichuriasis and hookworm infection (necatoriasis, ancylostomiasis), followed by schistosomiasis and lymphatic filariasis [1]. Some anthelmintic drugs have been developed and used for the treatment of helminthiases, but with some limitations. For example, anthelmintic drugs do not protect against reinfection, and treatment failure because of repeated use of these drugs has drawn attention to the development of drug resistance [6]. In addition, despite the promise of attenuated larvae, the use of which has shown promising results in vaccine studies, recombinant and epitope-based vaccine candidates show variable efficacy [2, 7, 8]. For example, a phase 1 clinical trial using ultraviolet-attenuated *Necator americanus* larvae elicited an antigen-specific humoral and cellular immune response that resulted in reduced larval output in vaccinated participants [8]. In contrast, recombinant protein-based vaccines have confirmed safety in humans, such as the glutathione-*S*-transferase of *Schistosoma haematobium*, but which was found to be ineffective in protecting from reinfection in a phase 3 study [9]. Despite all attempts, most vaccines against helminth infections remain trapped in early-stage development or are undergoing pre-clinical evaluation, and no human anti-helminth vaccine has yet been approved for widespread use [7, 10].

Identifying suitable protective antigens is one of the major challenges in vaccine design. This step relies on the availability of well-curated genomic, transcriptomic and proteomic databases, as well as on a detailed knowledge of helminth biology. Notwithstanding, the marked complexity as well as the limitations in availability and access to samples still represent a great handicap for basic research on helminth infections [11].

Progress in helminth biology research has gone hand in hand with the advances in techniques for genome sequencing and proteome characterisation. Helminth parasites have a large complex genome, ranging from approximately 42 to > 1000 Mb with up to 18,000 protein-coding genes [12]. The whole-genome shotgun (WGS) method was used to produce the first parasite genome, that of the filarial nematode *Brugia malayi*, in 2007 [13]. The rapid development of new sequencing platforms, such as Roche 454 sequencing system (Roche Diagnostics) and Illumina sequencing platforms (Illumina, Inc.) offered 1000-fold increase in throughput over the Sanger sequencing technology and supported the progressive publication of more helminth genomes [11]. Indeed, projects such as the 50 Helminth Genome Initiative have contributed to the list of 157 nematode and 45 platyhelminth genomes currently published at the WormBase ParaSite database [14]. As data on helminth genomes grew in magnitude, new transcriptomic and proteomic data were also released. In 2010, the complete transcriptome of the human liver fluke *Clonorchis sinensis* was reported which, soon after, gave place to proteomic studies that revealed the metabolic changes between aerobic and anaerobic pathways that evolved with the diverse niches colonised through the life-cycle [15]. The availability of these “omics” data has underpinned the development of transgenesis and genome-editing research, rendering key information on the molecular biology of helminths. From the early studies using biolistics and the microinjection of plasmids [16–18] to the recently deployed CRISPR/Cas9 (Clustered regularly interspaced palindromic repeats/CRISPR-associated) systems [19, 20] in nematodes and trematodes, these advances have contributed to a diverse array of pathways and processes, including the characterisation of immune responses and immunomodulatory molecules, as well as of the key proteins involved in haemoglobin digestion and tegument integrity in platyhelminths [21–24]. Despite the many steps forward, however, most of the helminth genomic data still consist of partial draft assemblies, and numerous “hypothetical” genes remain uncharacterised [25]. A profound comprehension of the applications and limitations of transgenesis available techniques will allow us to select the correct techniques as well as to explore the alternatives and improvements to widen our research possibilities and, thus, our knowledge on helminth biology. This review explores current knowledge, prospects and challenges for research on helminth biology through transgenesis techniques.

## First steps in helminth transgenesis

Countless advances in genomic research have been made since reports of the first targeted genomic changes produced in yeast and mice in the 1970s and 1980s, respectively. These advances have focussed on the reduction of the off-target effects, the enhancement of the efficiency and its application to different species [26].

Although transgenic lines have been established for numerous species, this feat has been achieved only in a few helminths. In addition to the associated obstacles of genome editing, research on helminths presents considerable obstacles in the context of genetic manipulation, due both to their biological complexity and to the requirements of parasitism [23]. For example, the life-cycle of most helminths consists of several developmental stages which are associated with morphological and biochemical changes that confer the ability to survive in different host/external environments. Furthermore, nematodes and platyhelminths frequently need intermediate hosts to complete their life-cycle, which makes their maintenance in the laboratory particularly challenging [27]. This complexity has traditionally hampered the establishment of stable transgenic lines in parasitic helminths. Not surprisingly, advances in the transgenesis of helminths occurred on the basis of *Caenorhabditis elegans* transgenesis, beginning in 1982 with the first successful transformation of this free-living nematode [28].

As important as selecting the appropriate genomic construct, providing the specific and highly effective technique for transfection is essential for programmed editing of the helminth genome. As new techniques have emerged, research possibilities have been expanded, but there are still imposing impediments to overcome in the research of different helminth species, as discussed in the following sections.

## Plasmid-based vectors

**Fig. 1:**
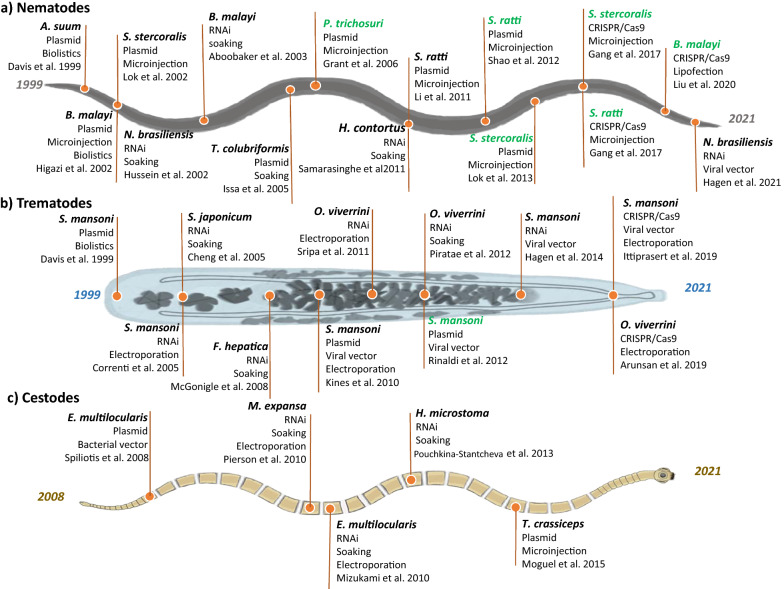
Historical advances in the transgenesis of parasitic helminths. The image shows the first successful transient/stable transgenesis of different species of nematodes (**a**), trematodes (**b**) and cestodes (**c**) using biolistics, microinjection, soaking, electroporation and lentiviral or bacterial vectors. Stable lines are highlighted in green. For full name of species, as well as reference number of citations, see Table 1

Early DNA constructs consisted of plasmid-based vectors that resulted in the assembly of transgene sequences into extrachromosomally inherited, multi-copy arrays, as shown in 1991 in the free-living model nematode, *C. elegans* [30]. Plasmids incorporate the gene of interest in combination with a selection marker, a transcriptional reporter and the corresponding cellular sequences for transcription and translation. In addition, the addition of shared homology sequences has allowed for the integration of these arrays into the chromosome [29, 31]. A wide range of delivery techniques have been applied over the years for plasmid-based transgenesis in the search for higher efficiency rates and stable lines. The first transient transfections of a fluorescent reporter transgene were accomplished in 1999 for the embryos of the parasitic nematode *Ascaris suum* and the adults of the trematode *Schistosoma mansoni* [16]. It was not until 2008 that the first successful transient transfection of primary cells of the cestode *Echinococcus multilocularis* was attained based on soaking [32]; this also opened the possibility to study the facultative intracellular bacterium *Listeria monocytogenes* as a method to introduce foreign DNA into *Echinococcus* cells. Spiliotis and co-authors reported the expression of the reporter gene by these bacteria after cytosolic lysis but without integration in the chromosomes of *E. multilocularis* (Fig. 1) [32].

sBiolistics-based transient transgenesis of *S. mansoni* revealed the tissue expression of the cathepsin F and B2 in the gut and tegument of this trematode, respectively [33]. This method was also applied to *B. malayi* and compared to microinjection, showing that the fluorescence activity of the microinjected parasites was higher than that seen in the biolistically transfected adults (Fig. 1; Table 1) [17]. Transfection mediated by microinjection of the cestode *Taenia crassiceps* was successful in expressing the GFP reporter under the control of a cytomegalovirus (CMV) promoter in 2015 (Fig. 1; Table 1) [34]. Considering these results and the high level of mortality among bombarded parasitic helminths, microinjection and other techniques generally have replaced biolistics in helminth plasmid-based transgenesis [29].

**Table 1:** First and key examples of transgenesis in nematodes, trematodes and cestodes of human and veterinary impact, the gene targeted and the methodology used

Three groups of parasitic intestinal helminths	Species	Plasmid				RNAi				CRISPR			
		Method	Gene	Date	References	Method	Gene	Date	References	Method	Gene	Date	References
Nematodes	*Ascaris suum*	Biolistics	*luciferase*	1999	[16]	Soaking	*enolase*	2011	[54]				
	*Haemonchus contortus*					Soaking	*H11 aminopeptidase*	2011	[53]				
	*Trichostrongylus colubriformis*	Soaking	*ubiquitin*	2005	[66]	Bacteria feeding	*tropomyosin*	2005	[66]				
	*T. colubriformis*	Electroporation	*ubiquitin*	2005	[66]								
	*Brugia malayi*	Biolistics	*GFP*	2002	[17]	Soaking	*β-tubulin*	2003	[52]	Lipofection	*GFP*	2020	[84]
	*B. malayi*	Microinjection	*GFP*	2002	[17]								
	*B. malayi*	Chemical transfectant	*luciferase*	2011	[45]								
	*Strongyloides stercoralis*	Microinjection	*GFP*	2002	[18]					Microinjection	*unc-22*	2017	[79]
	*S. stercoralis*	Microinjection	*GFP*	2006	[30]					Liposomes + Microinjection	*collagen*	2019	[83]
	*S. stercoralis*	Microinjection	*GFP*	2013	[39]								
	*Strongyloides ratti*	Microinjection	*GFP*	2011	[37]					Microinjection	*unc-22*	2017	[79]
	*S. ratti*	Microinjection	*GFP*	2012	[38]								
	*S. ratti*	Microinjection	*T cell epitope*	2021	[21]								
	*Nippostrongylus brasiliensis*					Soaking	*AChE B*	2002	[51]				
	*N. brasiliensis*					Viral vector	*β-tubulin/Ache B*	2021	[75]				
	*Parastrongyloides trichosuri*	Microinjection	*GFP*	2006	[35]								
Trematodes	*Schistosoma mansoni*	Biolistics	*luciferase*	1999	[16]	Electroporation	*cathepsin B*	2005	23]	Viral vector	*omega-1*	2019	[86]
	*S. mansoni*	Viral vector	*luciferase*	2006	[42]	Viral vector	*omega-1*	2014	[73]	Electroporation	*omega-1*	2019	[86]
	*S. mansoni*	Viral vector	*luciferase*	2008	[46]					Electroporation	*AChEs*	2021	[22]
	*S. mansoni*	Viral vector	*luciferase*	2012	[47]					Electroporation	*SULT-OR*	2021	[94]
	*S. mansoni*	Electroporation + viral vector	*luciferase*	2010	[43]								
	*S. mansoni*	Biolistics	*cathepsins ORF-GFP*	2010	[32]								
	*S. mansoni*	Electroporation	*cathepsins ORF-GFP*	2010	[32]								
	*S. mansoni*	Chemical transfectant	*mCherry*	2013	[44]								
	*S. mansoni*	Viral vector	*HIV-1 genes*	2016	[92]								
	*Schistosoma japonicum*					Soaking	*gynecophoral Canal Protein*	2005	[55]				
	*S. japonicum*					Microinjection	*cathepsin B1*	2018	[71]				
	*Opisthorchis viverrini*					Electroporation	*cathepsin B*	2011	[61]	Electroporation	*granulin*	2019	[20]
	*O. viverrini*					Electroporation + Soaking	*tetraspanins*	2012	[60]				
	*Fasciola hepatica*					Soaking	*cathepsin B & L*	2008	[56]				
	*F. hepatica*					Electroporation + Soaking	*Leucine aminopeptidase*	2014	[61]				
Cestodes	*Moniezia expansa*					Electroporation + Soaking	*actin*	2010	[59]				
	*Echinococcus multilocularis*	Bacterial vector	*CFP*	2008	[31]	Soaking	*elp*	2010	[62]				
	*E. multilocularis*					Electroporation	*elp*	2010	[62]				
	*Hymenolepis microstoma*					Soaking	*Hox*	2013	[63]				
	*Taenia crassiceps*	Microinjection	*GFP*	2015	[33]								

Heritable and stable transfection is readily obtained in *C. elegans* through the gonadal microinjection of plasmid-based vectors [35]. Techniques developed for *C. elegans* were conveniently adapted to parasites deploying at least one generation of free-living development, with adult males and females residing in soil contaminated by host-infected faeces [35]. In 2002, Lok and co-workers carried out the transfection of *Strongyloides stercoralis* by microinjection, but the expression of green fluorescent protein (GFP) was only detected on the maternal gonads and none of the transformed embryos hatched (Fig. 1; Table 1) [18]. Four years later, heritable transgenesis of the parasitic nematode *Parastrongyloides trichosuri* was achieved using microinjection (Fig. 1; Table 1) [36].

Early transgenesis research on *S. stercoralis* attempted the transgenesis with the collection of modular *C. elegans* transformation vectors which were designed to incorporate any promoter and coding sequence upstream of a multipurpose terminator called *unc*-54 3′untranslated region (UTR) [37]. However, it was not until a parasite-specific 3′UTR was incorporated that constructs were expressed in a sustained and tissue-specific pattern in developing larval and adult *Strongyloides* [37]. GFP from a plasmid-encoded transgene was detected by PCR in *S. stercoralis* transiently after microinjection, for at least five passages; however, the expression of this transgene could not be detected in transgenic worms from subsequent passages [37]. These investigators hypothesised that *S. stercoralis* gathered plasmid-encoded transgenes into tandem, multi-copy, episomal arrays that were inherited, but silenced after the first host passages due to their highly repetitive nature, their episomal location or both [38]. Similarly, initial plasmid-based transgenesis in *Strongyloides ratti* exhibited only transient expression of a fluorescent reporter (Fig. 1; Table 1) [39]. By integrating the transgene into the chromosome through the usage of the *piggyBac* (PB) transposon system, stable and heritable expression was achieved in 2012 for *S. ratti*, and 1 year later for *S. stercoralis* (Table 1) [31, 40]. The activity of PB in *S. mansoni* and integration of the transposon into schistosome chromosomes was reported several years earlier [41]. This system efficiently transposes between chromosomes and vectors via a “cut and paste” reversible activity of the PB transposase. The ability to integrate into the helminth genome made transposon and retrotransposons likely candidates as vectors to develop stable transfections [42].

The establishment of stable transgenic lines was possible initially in the well-adapted host parasite pairing of *S. ratti* in the rat host, which, added to the 50% efficiency of infection, has promoted the successful host passage of small numbers of transgenic iL3 in the first three generations of line establishment in 2012 (Table 1) [31]. Accordingly, the expression of a fluorescent reporter gene under the control of the actin promoter of *S. stercoralis* was uniformly detected for at least in 10 generations of passage (Table 1) [31]. The same protocol allowed for the transgenic expression of a specific T-cell epitope, which was able to constitute both Th2 and Treg populations from the initial helminth-activated CD4 + T cells, in *S. ratti*, unravelling novel features of the immune response to this nematode (Table 1) [21].

Subsequent relevant improvements were related to the potentially confounding effects of transgene overexpression from high-copy arrays, initially observed in *C. elegans,* which prompted the search for methods for low- or single-copy transgene integration into the chromosome [43]. A system for single-copy insertion of transgenesis mediated by the Mos1 transposon from *Drosophila mauritiana* was devised for *C. elegans* in 2012 [43]*.* When worms are subjected to heat shock, the transposase is expressed from the “transposase array” and catalyses the transposition of Mos1 from the “transposon array” into chromosome loci. Mos1 insertions represent molecular tags that are readily identified using inverse PCR [43].

Retroviral vectors alone or in combination with soaking or electroporation have also been used for the delivery of reporter transgenes in the transgenesis of helminths [44, 45]. The approach leverages, firstly, the essential nature of the life-cycle of this family of RNA viruses, specifically the proviral stage of the life-cycle that integrates as DNA into the genome of the host cell and, second, the pseudotyping of the virion with the vesicular stomatitis virus glycoprotein (VSVG), which expands the target host range. For example, Kines et al. accomplished transient luciferase expression in *S. mansoni* eggs using retroviral particles for plasmid delivery in 2006 (Table 1) [44], and soon thereafter in soaked schistosomula in Moloney murine leukaemia retrovirus (MLV) pseudotyped with VSVG, with the aim of delivering reporter transgenes into schistosome chromosomes [46]. Southern hybridisation analysis indicated the presence of proviral MLV retrovirus in the transduced schistosomes; subsequently, fragments of the MLV transgene and flanking schistosome sequences were recovered using an anchored PCR-based approach, thereby demonstrating definitively that somatic transgenesis of schistosome chromosomes had taken place and revealing widespread retrovirus integration of the provirus of this mammalian retrovirus into the schistosome chromosomes. In addition, these investigators also reported that more efficient transduction of schistosome eggs was feasible using square wave electroporation in tandem with soaking of schistosome eggs with the VSVG pseudotyped MLV virions (Fig. 1; Table 1) [45]. Notably, these researchers also reported germline transgenesis of *S. mansoni* following retroviral transduction of newly laid eggs, and the expression of a transgene encoding resistance to aminoglycoside antibiotics in the F1 progeny [47, 48].

As with biolistics, electroporation can lead to significant mortality, and this technique also requires the transfer of worms, prior to culturing, into cuvettes with nucleic acids in minimal salt solution to avoid arcing, which increases the possibility of contamination [49]. For these reasons, in 2013, Liang et al. proposed the use of chemical transfectants (e.g. polyethyleneimine) or co-precipitates in combination with the transgene DNA since these can improve the DNA uptake by promoting the binding to the cell surface and the subsequent endocytosis, as shown in schistosomes [49]. Additional successful transgenesis using this technique has been accomplished in *B. malayi* L3 [50]. These investigators suggested that exposing the infective stage filariae to a solution containing calcium phosphate and the transfecting plasmid DNA during a larval moulting cycle would increase the uptake possibly due to a higher catching ability of the nascent cuticle [50]. Indeed, various parasitic nematode species produce larvae that are able to undergo one or more complete moults in vitro or in accessible locations in mammalian or vector hosts [50].

## RNA interference

Plasmid-based vector techniques have paved the way for parasitic helminth transgenesis. Many limitations had been overcome through the single-copy insertion of the transgene into the chromosome, but random integration still represented a disadvantage for functional genomics since it could interfere with the expression of crucial genes for worm development and survival [31]. Furthermore, the production of stable intrachromosomal transgenic lines was also associated with high costs and intense workload. Consequently, much of the research effort to date has instead focussed on the downregulation of post-transcriptional gene expression through interfering RNAs using the RNA interference (RNAi) technique. RNAi is a very powerful tool that emerged from research on so-called non-coding RNAs (ncRNAs). These molecules vary not only in length (small and long ncRNAs with > 200 nt) but also in their biogenesis and roles in the cell [51]. A diverse variety of ncRNAs are known, but it is principally microRNAs (miRNA) that have attracted much attention due to their ability to inhibit mRNA translation by increasing their degradation or decreasing their stability. Similar to miRNA bioprocessing, researchers have managed to introduce exogenous long double-stranded RNAs (dsRNAs) that are cleaved in the cytoplasm by Dicer, rendering functional small interfering RNAs (siRNAs) which, as miRNAs, are able to promote the degradation of the targeted RNAs [51]. As demonstrated in *C. elegans*, RNA uptake and spreading starts in the intestine where the transmembrane transporter SID-1 is mainly expressed [52]. Likewise, a schistosome SID-1 homologue has been identified, suggesting that siRNAs initially accumulate faster in the gut and, considering the syncytial tissue of schistosomes, traverse large distances internally without the need to cross additional plasma membranes [53]. Additionally, siRNAs can be more efficient inhibitors when multiple oligonucleotides with distinct sequences are used against the same target [54]. Although it is possible to obtain siRNAs directly, dsRNA is more stable to RNase digestion and is more economical to produce than siRNAs [55]. For these reasons and the ease of RNA synthesis in comparison to protein synthesis, dsRNAs, designed for siRNA pathways, are still one of the most useful tools for the repression of gene expression in biology research [51].

Despite gene silencing by RNAi being successfully applied to a wide variety of organisms, including protozoa, amphibians, insects and a number of parasitic helminths infecting plants, animals and humans, RNAi has generated inconsistent and variable results between helminth species and between different genes within a species [56]. In comparison to nematodes (e.g. *Nippostrongylus brasiliensis* in 2002) and trematodes (e.g. *S. mansoni* in 2005), cestode research lagged due to the absence of robust reverse genetic methods until 2008 when *E. multilocularis* germinal cells were successfully transfected (Fig. 1; Table 1) [23, 32, 57].

Several single or combined delivery techniques have been tested over the last decades in the search for approaches to improve dsRNA uptake by helminths. In the free-living nematode *C. elegans*, RNAi can be effectively induced by simply soaking worms in dsRNA solution since this nematode possesses membrane channels (Systemic RNA Interference Defective [SID]) involved in the intestinal uptake of exogenous dsRNA into the cells (SID-1) and in the systemic spread of this dsRNA (SID-2) [53, 55]. In parasitic nematodes, soaking-based RNAi silencing transgenesis was first applied to *N. brasiliensis*, where soaking with acetylcholinesterase (AChE) B-specific dsRNA for 24 h gave potent suppression of all three secreted acetylcholinesterases (AChE A, B and C), but did not affect the motility (Fig. 1; Table 1) [57]. As a possible explanation, the authors hypothesised that the selected 5’ region of the *ache B* gene overlaps with the *A* and *C* isoforms but diverges from the neuromuscular AChEs. However, the conditions required for RNAi adversely affected the ability of *N. brasiliensis* to re-establish infections for further characterisation [57]. Soon after, Aboobaker and co-workers silenced the *β-tubulin* and RNA polymerase II large subunit genes of *B. malayi*, resulting in reduction of transcript levels and death of adult female nematodes in culture (Fig. 1; Table 1) [58]. Other successful silencing manipulations based on soaking helped researchers examine the role of gene products from parasitic nematodes such as *Haemonchus contortus* (H11 aminopeptidase) and *A. suum* (enolase), generating a significant reduction in worm burden and in worm length, respectively (Table 1) [59, 60].

In like fashion, trematodes were also analysed through silencing techniques based on soaking. For example, in 2005, Cheng et al. accomplished a 75% reduction in the transcript level of the gynecophoral canal protein of *Schistosoma japonicum* that, as established in previous studies, is expressed in the adult male worm and plays a critical role in the development of the male worm and in pairing with the female (Fig. 1; Table 1) [61]. Consistently, immunofluorescence results confirmed the inhibition of the expression of this protein in a dose-dependent manner [60]. Soaking-based silencing approaches also revealed the requirement for cathepsins (B and L) from the infective stage of *Fasciola hepatica* in the penetration of the rat intestinal wall since edited worms displayed a variety of movements, erratic locomotion and paralysis (Fig. 1; Table 1) [62]. The same approach revealed the role of tetraspanins in the maintenance of tegument integrity in the trematode *S. mansoni*, considering the thinner and more vacuolated tegument of modified worms compared to controls and, thus, provided a potential mechanism for the efficacy of tetraspanin-based vaccines against schistosomes (Table 1) [24]. For *F. hepatica,* Dell’Oca and co-workers also showed that a combined electro-soaking method gives persistent gene silencing for up to 3 weeks, opening the possibility to perform functional validations of therapeutic targets in vivo [63].

Recent large-scale RNAi screening analysis in *S. mansoni* has uncovered more than 250 potential therapeutic targets affecting neuromuscular function, tissue integrity, stem cell maintenance and parasite survival [64]. Furthermore, this genome-wide screen has provided information about the biochemical interferences produced by existing pharmacological agents. For example, the proteasome inhibitor ixazomib, as well as the inhibitor bortezomib, altered both the movement and attachment of schistosomes [65]. Additionally, the study of different inhibitors of the p97 protein, a component of the ubiquitin–proteasome system (UPS), as well as the long-term treatment with p97-specific RNAi, led to extensive tissue degeneration and death in vitro and, thus, exhibited the crucial role of the proteasome in *S. mansoni* survival [64].

Early RNAi-based experiments in cestodes studied the relative efficiencies of soaking and electroporation. In 2010, the successful knockdown of the actin transcript revealed the role of this protein in tegument integrity and the contractile ability of the ruminant cestode *Moniezia expansa* since worms exposed to act-1 dsRNA exhibited significant impairment of tegument formation and contractile ability (Fig. 1; Table 1) [66]. To avoid extended culture outside of the host, a combined soaking and electroporation method was used, generating a reduction of 71% in actin transcript levels [66]. Electroporation alone and in combination with soaking was applied in studies of the trematode *Opisthorchis viverrini*, generating decreased expression levels and decreased activity of cathepsin B (90%) and tetraspanin-1 (72%) [67, 68]. The resulting phenotypes, including the display of a distinctly vacuolated and thinner tegument in worms treated with dsRNA targeting tetraspanin-1, exposed a crucial role of the targeted gene in tegument integrity in *O. viverrini*. (Fig. 1; Table 1) [67]. Notwithstanding, silencing studies in the cestode *E. multilocularis* showed that 10 min of electroporation was sufficient for the introduction of siRNA (72% efficiency), while soaking alone reached lower silencing levels (22%) (Fig. 1; Table 1) [69]. In contrast, delivery of target-specific dsRNA by soaking to the cestode *Hymenolepsis microstoma*, resulted in 80% suppression of the Hox transcription factor (Fig. 1; Table 1) [70]. However, the limited growth achieved prevented the characterisation of potential loss of function phenotypes in this study [70].

The high efficiency obtained with electroporation led to a progressive increase in the use of this method, while other delivery techniques were also tested. The successful transient transfection of *S. mansoni* using a luciferase-encoding RNA via electroporation in 2004 opened the possibility to apply the RNAi technology more widely [71]. Consequently, the same investigators proceeded to test electroporation-based silencing of the cathepsin B gene. Resulting phenotypes, including growth retardation without loss of the gut heme pigmentation, highlighted the crucial role for this protein in the normal growth of this parasite [23]. Indeed, suppression of target mRNA was tenfold greater here than in previous studies utilising the soaking method [23].

Nonetheless, the application of electroporation has been far less successful in nematodes than in parasitic platyhelminths. The main limitation appears to be the low permeability of the non-cellular nematode cuticle, which differs from the syncytial tegument of trematodes and cestodes [72]. In larval and adult *N. brasiliensis*, only partial and inconsistent knockdown of transgene expression was achieved. Consequently, this method does not provide a viable means of delivery in nematodes such as *N. brasiliensis* [72].

Alternatively, feeding of bacteria engineered to express dsRNA has been used very effectively for dsRNA delivery in *C. elegans*, although it was not effective when *Escherichia coli* expressing dsRNA was tested for *N. brasiliensis, H. contortus* and *Heligmosomoides polygyrus* [72]. It is also necessary to consider that infective larvae are developmentally arrested in strongylid nematodes and do not feed, presenting a problem in terms of dsRNA delivery [72]. In this regard, chemical exposure and maintenance at elevated temperature can enhance feeding [73–75]. Resorcinol and octopamine stimulate uptake of dsRNA by plant-parasitic nematodes [74, 75], and exposure of *N. brasiliensis* L3 to 37 ºC for 4 h proved sufficient to stimulate feeding, although inconsistent knockdown results were obtained [73].

Subsequent to the first successful application of RNAi by microinjection being achieved in 2010 in the (free-living) planaria of the genus *Dugesia* [76], this delivery technique has been widely used, generally for silencing experiments in platyhelminths. The possibility of performing gene functional analysis by RNAi led to the isolation of elements involved in the control of patterning and axial polarity during planarian regeneration and homeostasis [76]. Microinjection-based silencing has been applied to nematodes, such as *Pristionchus pacificus*, and to the blood fluke *S. japonicum*, generating a non-rolling F1 progeny and showing the crucial role of cathepsin B1 in the digestion of haemoglobin, respectively (Table 1) [77, 78]*.* Furthermore, by microinjecting the dsRNA into the tail vein of infected mice, a highly effective and specific method for suppressing the gene encoding cathepsin B1 expression in vivo was established for *S. japonicum* [77]. The reduced expression of cathepsin B1 resulted in less heme pigmentation in the intestinal tissue, reflecting reduced haemoglobin digestion, which explains the growth retardation of schistosomula in mice [77]. After the successful transient plasmid-based transfection of *T. crassiceps* in 2015, Moguel et al. continue to explore delivery strategies for the introduction of dsRNA and the generation of stable transfection in *T. crassiceps*, including the PB transposon [49].

Recently, helminth transgenesis has deployed pseudotyped retroviral vectors to explore new methods to obtain higher transformation efficiency [29, 79]. Specifically, a lentivirus-based transduction system containing miRNA-adapted short hairpin RNAs (shRNAmirs) was employed in 2014 for the knockdown of the *omega-1* gene in *S. mansoni* eggs, which significantly decreased the volume of circumoval granuloman mice infused intravenously with these eggs (Fig. 1; Table 1) [80]. Short hairpin RNAs (shRNAs) are artificial RNA molecules synthesised within the cell by DNA vector-mediated integration. They consist of two complementary 19- to 22-bp RNA sequences linked by a short loop of 4–11 nt that is similar to the hairpin found in cellular miRNA [81]. Ultimately, the success of transformation relies on the susceptibility of the schistosome’s tegument to lentivirus transduction which, as mentioned for electroporation, presents similar difficulties for its application in nematodes. Nevertheless, the epithelial layer of the nematode gut may allow the entry of pseudotyped viruses [29, 79]. Indeed, the recent transfer of lentiviral vectors encoding shRNAmers into the nematode *N. brasiliensis* has been successfully obtained through the feeding of third-stage larvae (L3) with lentiviral particles that entered via the intestine and transcribed siRNAmers in eggs and infective larvae (Fig. 1; Table 1). Nonetheless, no obvious phenotypic effect after silencing any of the targets (*β-tubulin isotype-1* and secreted *acetylcholinesterase B*) were observed in L3 in vitro*,* possibly due to the modest knockdown of target mRNA achieved and the lack of shRNAmer expression in adult worms (Table 1) [82]. The authors hypothesised that the lack of transcription could be due to chromatin-mediated transcriptional silencing of the viral integration site during parasite development. Further optimisation of the expression cassette is needed to overcome the problem of transcriptional silencing during development [82].

## Advanced techniques: CRISPR/Cas9

As noted, RNAi-based gene silencing has helped reveal some gene functions crucial for studying the biology of helminths of clinical and veterinary importance. However, RNAi often produced undetectable or subtle phenotypic changes and was either transient or the inheritance of gene silencing was not fully penetrant [83]. The establishment of a highly specific technique for the direct knockout of the target gene has been fundamental to overcome these limitations. This has now become feasible with the arrival of CRISPR technology [84]. After the first successful gene editing in mammalian cells in 2013 with CRISPR [85], this technology has found broad application in fields ranging from the food industry to human health [86].

The chromosomes of prokaryotic species can contain from one to eight CRISPR loci consisting of: (i) direct repeats bordered with spacers; (ii) leader sequence adjacent to the CRISPR array; and (iii) CRISPR-associated (*cas*) genes coding for Cas proteins (93 different *cas* genes have been identified until now) [86]. In brief, the system consists of an RNA-guided nuclease that introduces insertions or deletions in target genomic sites [86]. The most widely used version of this approach involves mutagenesis catalysed by a complex of the *Streptococcus pyogenes* Cas9 and a small guide RNA [87]. Nonetheless, commercially available variants of this system involve using a deactivated form of Cas9 to tether activator (CRISPRa) or repressor protein domains (CRISPRi) to the target sequence for the purpose of enhancing or supressing target gene expression, respectively [87].

To date, the CRISPR/Cas9 system has been successfully applied in free-living nematodes, parasitic nematodes and trematodes through microinjection, electroporation or lentiviral-mediated transduction [83]. However, to our knowledge, CRISPR-based genome editing studies have not yet been reported for cestodes, likely because of the requirement for the identification and isolation of germinal cells as well as the development of a procedure for the stable transfection [34]*.*

Many targeted mutagenesis successes have been reported for *S. stercoralis,* providing more details about the biology of this organism and opening the possibility of applying this technique in related genera of parasites [29]. Mutations in *S. stercoralis* as well as in *S. ratti* have been created by transducing with basic CRISPR elements and selectable markers encoded in plasmid vectors or by microinjecting gonads of free-living females with pre-formed nucleoproteins of a recombinant Cas9 and in vitro transcribed genomic RNAs [88]. In *S. stercoralis* and *S. ratti,* the disruption of the twitchin protein (*Ss-ucn-22* gene), a large intracellular muscle protein homologous to mammalian connectin, generated free-living adults and infective larvae with severe motility defects (Fig. 1; Table 1) [88, 89]. Notwithstanding, the first-generation larvae were heterozygous for the CRISPR/Cas9-mediated insertion. Consequently, the next paramount goal was to adapt the CRISPR/Cas9 system in *Strongyloides* spp. for rendering the mutations homozygous [29]. Considering the hermaphroditism of *C. elegans*, homozygous lines for the mutation were derived easily within a single second generation for subsequent rearing, genotyping and phenotypic analysis [29]. The relatively low efficiency of *S. stercoralis* infection in the gerbil, as well as the lack of homozygous first-generation larvae, for both *S. stercoralis* and *S. ratti* initially hampered attempts to obtain stable transgenic lines [29]. In 2017, Gang et al. overcame this limitation, obtaining homozygous deletions of the *twitchin* gene *unc*-22 in approximately 2–5% of the F1 infective L3s in both *S. stercoralis* and *S. ratti* microinjected with plasmid vectors or ribonucleoprotein particle (RNP) complexes into the syncytial gonads [88]. Comparatively, transduction of parental worms with plasmid-encoded CRISPR/Cas9 elements has produced more efficient mutagenesis than transduction with pre-formed nucleoproteins. As in *C. elegans*, the deletion of *twitchin*, which encodes a large intracellular muscle protein homologous to mammalian connectin, causes an uncoordinated phenotype being more frequent in *S. stercoralis* than in *S. ratti*. The authors also suggested the improvement of the strategy by targeting both free-living males and females to improve the incidence of F1 homozygous knockouts [86]. Subsequent CRISPR/Cas9-mediated homozygous disruption methods have helped link some *S. stercoralis*-specific genes to functions involved in host infection (disruption of *Ss-tax-4* severely affects positive thermotaxis toward host body temperatures) [88] and, recently, to development (disruption of DAF-12 and DIP-1 proteins are crucial for the developmental arrest and reactivation of infectious L3, respectively) [91, 92].

The potential off-target sites, the high variability in temporal patterns of expression and the difficult selection of appropriate tissue-specific promoters and other regulatory elements have created the need for alternative CRISPR/Cas9 approaches. The need to express Cas9/single guide RNA (sgRNA) from transgenes has been obviated in the free-living nematodes *C. elegans* and *P. pacificus* by using pre-formed nucleoproteins in 2013 and 2015, respectively [19, 93]. Cas9 has been commercialised in a lyophilised form, and is ready to be combined with synthetic sgRNAs to be introduced, for example, into the syncytial gonads of the worms by microinjection. The transient activity renders mutant progeny produced within 9 h of microinjection in *P. pacificus* [19, 94]. In addition, heterozygous mutations in both helminths could be rendered homozygous by self-fertilisation and crossing of worms cultured through multiple generations [29]. Recently, Adams and co-workers combined liposome-based technology with microinjection to produce the CRISPR-mediated mutagenesis in the nematodes *Auanema rhodensis* (free-living) and *S. stercoralis* (parasitic) [95]*.* The authors accomplished the mutagenesis of *A. rhodensis* for the first time, obtaining a right-handed roller phenotype even in the F2 generation following dsRNA administration. However, the impaired progressive motility of roller mutants of *S. stercoralis* hindered host passage of mutant larvae to ascertain their infectivity and heritability [95]. CRISPR-mediated transfection in nematodes also has been carried out in *B. malayi*, successfully integrating the luciferase reporter in the genome of this species with no off-target insertions found and, thus, opening the possibility to precisely edit the genome of this important filarial parasite [89, 96].

In addition, the study of genetic mutations or transcriptional alterations that disable essential functions for invasion, migration and infection establishment was impractical before researchers started to apply conditional expression [29]. Although temporal as well as anatomical expression patterns depend upon the specific promoter, the possibility to control or regulate the expression of the gene of interest would help, especially in helminth biology research, since it could open the possibility to study pre-parasitic larvae and the biology and host interactions of post-infective larvae and parasitic worms.

Many alternatives for the conditioning of transgene expression have been applied. Regulation at the transcriptional level was attained by using promoters with tetracycline-regulated motifs, while for post-translational regulation researchers used recombinant proteins fused to degradation domains which can be stabilised in the presence of specific analogues. This latter system has proven effective in the malaria parasite *Plasmodium falciparum* through the design of a recombinant protein fused to the degradation domain of *E. coli* dihydrofolate reductase (DHFR-DD). This domain is stabilised in the presence of the folate analogue antibiotic trimethoprim (TMP), avoiding proteasome degradation. In addition, this system has also been successfully applied to *S. stercoralis* larvae for the regulatable expression of GFP in the presence of TMP [29]. Additionally, the Cas9 nuclease can be expressed in two fragments tethering the FK506-binding protein 12 (FKBP) and the FKBP-rapamycin binding (FRB) domains, respectively. These fragments combine to reconstitute the active nuclease in the presence of the drug and, thus, target specific genomic sites by appropriate sgRNAs. Another advantage of this system is the lower indel frequencies in validated on- and off-target sites when compared to wild-type Cas9. Lastly, a split inactive Cas9 was also used for transcriptional activation by the fusion of the FKBP fragment to the VP64 transcriptional activator [97].

In trematodes, at the end of the 2010s, CRISPR/Cas9 was used to mediate the knockout of the *granulin* gene in *Opisthorchis viverrini* through electroporation (Fig. 1; Table 1) [20, 89]. This liver fluke represents the principal risk factor for cholangiocarcinoma (CCA) in countries such as Thailand [98, 99]. The mutation resulted in reduced pathology when gene-edited parasites colonised the biliary tract of hamsters [20]. In a recent preprint, these same investigators appeared to confirm a role for liver fluke granulin in malignant transformation of the biliary tract [100]. Following upon the findings with VSVG pseudotyped MLV, VSVG pseudotyped HIV-1 (human immunodeficiency virus 1) lentiviral virions was also reported to facilitate CRISPR-based transfection of schistosomes [80, 89] and to deliver proviral transgenes into the chromosomes of *S. mansoni* [101]. For example, in 2019, Ittiprasert and co-workers tested this technique to edit the *omega-1* gene of *S. mansoni* via electroporation or by transduction with lentiviral particles (Fig. 1; Table 1) [102]. Similar efficiency rates for both techniques were reported. Notably, phenotypes resulting from disruption of *S. mansoni* omega 1 demonstrate the crucial roles of this ribonuclease in the polarisation of Th2 cytokine responses and in the formation of pulmonary granulomas surrounding the eggs of this schistosome. These host responses were significantly reduced in the presence of mutated eggs [102]. Recently, Ittiprasert and co-workers also demonstrated that a second programmable nuclease, Cas12a, also was active in *S. mansoni*; in a side-by-side comparison targeting the *omega-1* gene, Cas12a exhibited significantly higher efficiency than Cas9 for programmed knockout of this egg-stage expressed enzyme [103].

Soon after the appearance of the initial report by Ittiprasert and colleagues confirming the activity of Cas9 in schistosomes [99], Rinaldi and co-authors induced CRISPR/Cas9-mediated deletion mutations in the *SULT-OR* gene that encodes a sulfotransferase expressed in the parasitic stages of *S. mansoni*; this gene is required for anthelmintic activity of oxamniquine against *S. mansoni* [104]. Divergent mutagenic efficiencies were observed between different developmental stages, with adults shown to be the most susceptible stage to programmed deletions in the *SULT-OR* gene, yet knockdown at the mRNA level was not observed [104]. Therefore, researchers keep working on further optimisation of CRISPR-Cas protocols for the generation of a homozygous knockout in the *SULT-OR* gene [104]. Parallel experiments conducted by You et al. showed that the CRISPR/Cas9 technology also enables the knockin into the genome of the eggs of *S. mansoni* via electroporation [22]. These authors edited the AChE gene, introducing stop codons in exons 5 and 7 (Table 1), which revealed the potential immunomodulatory function of the domain located between these exons, since edited eggs elicited an increased Th2 response characterised by elevated levels of interleukin (IL)-4, IL-5, IL-10 and IL-13 [22]. Finally, Hulme et al. employed both RNAi and programmed gene editing approaches in parallel to demonstrate that the glycosyl hydrolase *S*. *mansoni* α-*N*-acetylgalactosaminidase (SmNAGAL) plays a central role in the cellular machinery of glycan processing and modification, with dysregulation of coordinated parasite movement and egg production following both gene silencing or gene knockout of SmNAGAL [105].

## Helminth transgenesis: current gaps and future perspectives

Advances in general genome manipulation have reached, to some degree, research initiatives on parasitic helminths, opening new possibilities for understanding the basic biology of these organisms and, thus, uncovering more potential therapeutic targets. However, genome manipulation in parasitic helminths still lags behind the current state-of-the-art capabilities for many other organisms. This is, in part, due to the specific complexities of parasitic helminths and their sophisticated life-cycles and the associated biochemical changes they deploy as they move between the free-living and parasitic environments.

Current methodologies have allowed the generation of transient expression, although these do involve an intensive workload and high cost and have variable success depending on the delivery method. Transient expression has resulted in the publications of a number of studies based on gene tagging and functional disruption. However, extended genomic research requires the establishment of stable transgenic lines to determine targets crucial for life-cycle development, host infection and parasite survival. All things considered, plasmid-based vectors helped to generate the first stable transgenic lines in some nematodes and trematodes [21, 31, 40, 47, 48], and CRISPR has also shown potential for the generation of transgenic lines in nematodes [88, 96] (Additional file 1: Table S1); however, reliable methods of delivering transgenes to the germlines of helminths need to be developed and established for most helminths.

Additionally, improvement of current transgenic tools, either through combining methodologies or refining current methods, will result in increased knowledge of helminth biology. For example, replacement of the genomic regulatory elements in transformation constructs has increased transgenesis efficiency. Indeed, substitution of the HCMV promoter by the hlh11 promoter of *C. elegans* and the addition of the tbb2 3’UTR in the vector used for the transgenesis of *N. brasiliensis* infective larvae produced a combined tenfold increase in its silencing capacity [82]. Additionally, the efficiency of a delivery approach may differ for each of the parasite life-stage and genes. Thus, several methods, alone or combined, must be tested. As an example, L1–L3 of *H. contortus* differ in their susceptibility to silencing depending on the method used: soaking-based silencing of the β-tubulin gene only renders phenotypic changes in the L3 but is ineffective for other genes in the same stage; the feeding method is not effective; and electroporation is effective in reducing transcript levels in L1 but larval death was observed [106–108].

Ideally, specific changes with stronger and sustained phenotypic effects will facilitate conclusive findings in helminth biology. However, limitations, such as off-target effects, can affect these phenotypic changes, and attention should be paid when applying any silencing and transgenesis technique. After all, the possibility to activate the cellular homology directed repair (HDR) processing providing a donor template with homologous ends, in conjunction with the variable collection of Cas9 formats, established CRISPR as the most specific, stable and flexible technique for genome editing. Despite CRISPR-based knockout experiments in helminths being initially limited to genes that are non-essential for worm survival, conditional transgene expression or regulable Cas9 activation has recently opened the possibility to interfere/disrupt these genes at the appropriate time. Nonetheless, for experiments based on a progressive loss-of-function, RNAi has demonstrated great potential since the resulting silencing level usually is dose-dependent, as shown in the silencing of the gynecophoral canal protein of *S. japonicum* [61]. Thus, although RNAi cannot completely knockout target genes, it can be useful to probe the functions of genes that are essential for parasite survival.

## Conclusions

Much time has passed since the first genome sequencing technique appeared and substantial amounts of data on the biology of other organisms have accumulated, but research on helminths still faces important limitations, and extensive “omics” analyses are needed to deeply understand the nature of parasitism. Here, we have summarised the available techniques in genome editing for biological research on helminths. Different genome constructs as well as different carriers have been tested for transgenesis in helminths with variable or inconsistent results.

The most promising results have been attained through the conditional expression of the transgene since it allows for the study of pre- and post-infective stages. However, not only the initiation but also the regulation and the end of the expression need to be controlled to achieve a feasible system for the study of molecular biology in helminths. Consequently, genomic sites for DNA integration that do not go through chromatin silencing need to be identified at least in the developmental stages of the helminth under study and, ideally, the whole life-cycle. Thus, the activation, regulation and silencing of transgenes would be possible in any stage of the helminth, providing a flexible system for research.

Notwithstanding, this is far from the current research capacity since transgenesis is still facing methodological limitations, with robust culture and delivery methods remaining among the principal limitations. Even the recent and promising advanced technique, CRISPR/Cas, requires improved CRISPR systems delivery and transgenesis efficiency as well as measures to minimise undesired off-target events to successfully and securely edit genomes. With the current platforms in place, ambitious projects in helminth research will benefit from this technological progress. Establishing collaborations, the development, modification and delivery of technical advances and continued improvements in the quality of curated genomes applicable to the targeted helminth species can be expected to be repaid with notable step changes in this field. In turn, it is hoped that these advances will enable improved prognosis, diagnosis and treatments for the helminthiases of global public health and economical significance.

## Supplementary Information

**Additional file 1: Table S1.** Extended details in the historical advances in the transgenesis of parasitic helminths.

## Data Availability

Not applicable.

## Abbreviations

AChE: Acetylcholinesterases
Cas: CRISPR-associated
CCA: Cholangiocarcinoma
CMV: Cytomegalovirus
CRISPR: Clustered regularly interspaced palindromic repeats
DALYs: Disability-adjusted life years
DHRF-DD: Degradation domain of the dihydrofolate reductase
dsRNAs: Double-stranded RNAs
FKBP: FK506-binding protein
GFP: Green fluorescent protein
HIV-1: Human immunodeficiency virus 1
HDR: Homology directed repair
miRNAs: MicroRNAs
MLV: Murine leukaemia retrovirus
ncRNAs: Non-coding RNAs
PB: *Piggyback*
RNAi: RNA interference
STHs: Soil-transmitted helminths
sgRNA: Single guide RNA
shRNAs: Short hairpin RNAs
shRNAmirs: MiRNA-adapted short hairpin RNAs
SID: Systemic RNA interference defective
siRNAs: Small interfering RNAs
TMP: Trimethoprim
VSVG: Vesicular stomatitis virus glycoprotein
WGS: Whole-genome shotgun
